# Supplementary material for: Flagellum and toxin phase variation impacts intestinal colonization and disease development in a mouse model of Clostridioides difficile infection
Source: Gut Microbes. 2022 Feb 22;14(1):2038854. doi: 10.1080/19490976.2022.2038854 (PMC8890394; doi:10.1080/19490976.2022.2038854)
Supplement: Supplemental Material [file KGMI_A_2038854_SM5851.zip › supplementary/downloadFromZipFile 1.pdf]

Table S1. Strains and plasmids used in this study

***Clostridioides difficile* strains**			
**Lab Notation**	**Strain Name**	**Description**	**Reference**
RT273	*C. difficile* R20291	Ribotype 027 strain	1
RT1693	*recV flg* OFF	R20291 *recV::ermB* (*flgB* UTR^{OFF})	2, 3
RT1702	*recV flg* ON	R20291 *recV::ermB* (*flgB* UTR^{ON})	2
RT1566	*sigD*	R20291 *sigD::ermB*	2
RT1658	*spo0A*	R20291 *spo0A::ermB*	4
RT2555	*flgB*ΔUTR	R20291 *flgB*ΔUTR	This work
RT2603	*flg*-3sub OFF	R20291 *flg*-3sub OFF	This work
RT2604	*flg*-ΔRIR OFF	R20291 *flg*-ΔRIR OFF	This work
RT2609	*flg*-Δ3 OFF	R20291 *flg*-Δ3 OFF	This work
RT2610	*flg*-3sub ON	R20291 *flg*-3sub ON	This work
RT2611	*flg*-ΔRIR ON	R20291 *flg*-ΔRIR ON	This work
RT2612	*flg*-Δ3 ON	R20291 *flg*-Δ3 ON	This work
RT2196	WT vec	R20291 with pRT1611 (vector control)	5
RT1562	WT pRecV	R20291 with pRT1611::*recV*	This work
RT2614	*flg*-3sub ON vec	R20291 *flg*-3sub ON with pRT1611	This work
RT2615	*flg*-3sub ON pRecV	R20291 *flg*-3sub ON with pRT1611::*recV*	This work
RT2616	*flg*-ΔRIR ON vec	R20291 *flg*-ΔRIR ON with pRT1611	This work
RT2617	*flg*-ΔRIR ON pRecV	R20291 *flg*-ΔRIR ON with pRT1611::*recV*	This work
RT2618	*flg*-Δ3 ON vec	R20291 *flg*-Δ3 ON with pRT1611	This work
RT2619	*flg*-Δ3 ON pRecV	R20291 *flg*-Δ3 ON with pRT1611::*recV*	This work

***Escherichia coli* strains**			
**Lab Notation**	**Strain Name**	**Description**	**Reference**
	Escherichia coli DH5α	F- φ80*lacZ*ΔM15 Δ(*lacZYA*-*argF*)U169 *recA1 endA1 hsdR*17(rκ -, mκ+) *phoA supE44 thi*-1 *gyrA96 relA1* λ- *tonA*	Invitrogen 6
RT270	*Escherichia coli* HB101(pRK24)	*E. coli* used in conjugations with *C. difficile*, Amp^R, Cm^R	7
RT1310	pFlg OFF pRecV (Ec)	DH5α co-transformed with pRT1324 and pRT1164	2
RT2587	pFlg-3sub OFF pRecV (Ec)	DH5α co-transformed with pRT2582 and pRT1164	This work
RT2588	pFlg-ΔRIR OFF pRecV (Ec)	DH5α co-transformed with pRT2583 and pRT1164	This work
RT2589	pFlg-Δ3 OFF pRecV (Ec)	DH5α co-transformed with pRT2584 and pRT1164	This work
RT2602	pFlg ON pRecV (Ec)	DH5α co-transformed with pRT1323 and pRT1164	This work
RT2590	pFlg-3sub ON pRecV (Ec)	DH5α co-transformed with pRT2585 and pRT1164	This work
RT2593	pFlg-ΔRIR ON pRecV (Ec)	DH5α co-transformed with pRT2592 and pRT1164	This work
RT2591	pFlg-Δ3 ON pRecV (Ec)	DH5α co-transformed with pRT2586 and pRT1164	This work

**Plasmids**			
**Lab notation**	**Plasmid Name**	**Description**	**Citation**
pRT709	pRPF185	pMTL960-derivative, contains ATc-inducible P*tet* promoter	8
pRT1611	pRPF185 EV	*gusA* removed from pRPF185	2
pRT1529	pRecV	pRT1611::*recV*	2
pRT1391	pMWO-074	Low copy vector with ATc-inducible P*tet* promoter	9
pRT1164	pRecV (Ec)	pMWO-074::*recV*	2
pRT2460	pMSR0	*E. coli-C. difficile* shuttle vector for toxin-mediated allele exchange mutagenesis	10
pRT2546	pMSR0 *flgB*ΔUTR	pMSR0 with construct for *flgB* UTR deletion	This work
pRT2570	pMSR0 *flg*-3sub OFF	pMSR0 with construct for *flg*-3sub OFF	This work
pRT2571	pMSR0 *flg*-ΔRIR OFF	pMSR0 with construct for *flg*-ΔRIR OFF	This work
pRT2572	pMSR0 *flg*-Δ3 OFF	pMSR0 with construct for *flg*-Δ3 OFF	This work
pRT2573	pMSR0 *flg*-3sub ON	pMSR0 with construct for *flg*-3sub ON	This work
pRT2580	pMSR0 *flg*-ΔRIR ON	pMSR0 with construct for *flg*-ΔRIR ON	This work
pRT2574	pMSR0 *flg*-Δ3 ON	pMSR0 with construct for *flg*-Δ3 ON	This work
pRT264	pMC123	pMC-P_{cpr} with nisin-inducible *cpr* promoter	11
pRT1324	pFlg OFF	pMC123::*flg* OFF	2
pRT2582	pFlg-3sub OFF	pMC123::*flg*-3sub OFF	This work
pRT2583	pFlg-ΔRIR OFF	pMC123::*flg*-ΔRIR OFF	This work
pRT2584	pFlg-Δ3 OFF	pMC123::*flg*-Δ3 OFF	This work
pRT1323	pFlg ON	pMC123::*flg* ON	2
pRT2585	pFlg-3sub ON	pMC123::*flg*-3sub ON	This work
pRT2592	pFlg-ΔRIR ON	pMC123::*flg*-ΔRIR ON	This work
pRT2586	pFlg-Δ3 ON	pMC123::*flg*-Δ3 ON	This work

References

1. Stabler RA, He M, Dawson L, et al. Comparative genome and phenotypic analysis of *Clostridium difficile* 027 strains provides insight into the evolution of a hypervirulent bacterium. *Genome Biol.* 2009;10(9):R102.
2. Anjuwon-Foster BR, Tamayo R. A genetic switch controls the production of flagella and toxins in *Clostridium difficile*. *PLoS Genet.* Mar 2017;13(3):e1006701.
3. Sekulovic O, Ospina Bedoya M, Fivian-Hughes AS, Fairweather NF, Fortier LC. The *Clostridium difficile* cell wall protein CwpV confers phase-variable phage resistance. *Mol Microbiol.* 2015;98(2):329-342.
4. Edwards AN, Nawrocki KL, McBride SM. Conserved oligopeptide permeases modulate sporulation initiation in *Clostridium difficile*. *Infect Immun.* 2014;82(10):4276-4291.

5. Garrett EM, Sekulovic O, Wetzel D, et al. Phase variation of a signal transduction system controls *Clostridioides difficile* colony morphology, motility, and virulence. *PLoS Biol.* Oct 2019;17(10):e3000379.
6. Hanahan D. Studies on transformation of Escherichia coli with plasmids. *J Mol Biol.* 1983;166(4):557-580.
7. McBride SM, Sonenshein AL. Identification of a genetic locus responsible for antimicrobial peptide resistance in *Clostridium difficile*. *Infect Immun.* 2011;79(1):167-176.
8. Fagan RP, Fairweather NF. *Clostridium difficile* has two parallel and essential Sec secretion systems. *J Biol Chem.* 2011;286(31):27483-27493.
9. Obrist MW, Miller VL. Low copy expression vectors for use in *Yersinia* sp. and related organisms. *Plasmid.* Jul 2012;68(1):33-42.
10. Peltier J, Hamiot A, Garneau JR, et al. Type I toxin-antitoxin systems contribute to the maintenance of mobile genetic elements in *Clostridioides difficile*. *Commun Biol.* Nov 27 2020;3(1):718.
11. Purcell EB, McKee RW, Courson DS, et al. A nutrient-regulated cyclic diguanylate phosphodiesterase controls *Clostridium difficile* biofilm and toxin production during stationary phase. *Infect Immun.* 2017;85(9).
