# Supplementary material for: Flagellum and toxin phase variation impacts intestinal colonization and disease development in a mouse model of Clostridioides difficile infection
Source: Gut Microbes. 2022 Feb 22;14(1):2038854. doi: 10.1080/19490976.2022.2038854 (PMC8890394; doi:10.1080/19490976.2022.2038854)
Supplement: Supplemental Material [file KGMI_A_2038854_SM5851.zip › supplementary/downloadFromZipFile 2.pdf]

Table S2. Oligonucleotides used in this study

Lab Notation	Primer name	Sequence (5’ to 3’)[a]	Reference
R850	rpoCqF	CTAGCTGCTCCTATGTCTCACATC	[39]
R851	rpoCqR	CCAGTCTCTCCTGGATCAACTA	[39]
R2175	flg_switchON_qF	GTTTTCTTACCAAAGTGATACATTATTATATTAATG	[23]
R2176	flg_switchOFF_qF	CATTAATATAATAATGTATCACTTTGGTAAGAAAAC	[23]
R2177	flg_switch_qR	GCTATTGTCTGACTTCTTAAATTAGTTGCAT	[23]
R2313	flg_switch_F	ATCACATTATGTAGTAAAAAACACC	This work
R2314	flg_switch_R	GTATTCACACTCACTCCTCC	This work
R2462	flgON_RIR_F	ATATTAGTTTTCTTACCAAAGTGATAC	This work
R2463	flgOFF_RIR_F	TAATAATGTATCACTTTGGTAAGAAAAC	This work
R3166	flgBqRb	TGGCATAGCATCATTTAATGTTTCTTC	This work
R1614	CDR20291_0248InvF	AGGCAACTTTATAAAGAAATATTTAAATTTATATTAAAATATTTTTATATTTTTATTAGG	[23]
R1615	CDR20291_0248InvR	CCTAATAAAAATATAAAAATATTTTAATATAAATTTAAATATTTCTTTATAAAGTTGCCT	[23]
R2459	flgB_UTR_F1	CAGGAAACAGCTATGACCGCGGCCGCGATTGTGCTCAATCTCATGG	This work
R2448	flgB_UTR_R1	CACACTCACTCCTCCTCACTATTTAGTTTTAACTTAAGTATACAATAAATAAC	This work
R2449	flgB_UTR_F2	GTTATTTATTGTATACTTAAGTTAAAACTAAATAGTGAGGAGGAGTGAGTGTG	This work
R2450	flgB_UTR_R2	GATCGCGCATGTCTGCAGGCCTCGAGCTTATCAACTTCTGTTCTAGGTAC	This work
R2672	flgBΔUTR_screen_F	GGAGATGCAGGAGCTATAG	This work
R2741	flgBΔUTR_F1	CATTGATTTCTTTCAGTTTCGGATCCTTGTGCTCAATCTATGGG	This work
R2742	flgBΔUTR_R2	GACGTCGACTCTAGAGGATCCCATAGATAGCTGTGCTTCTTGACC	This work
R2743	pMSR0_screen_F	GTGTTATCAATTGCACTACTCATGG	This work
R2744	pMSR0_screen_R	GTTGAACCATTAGCTAAGGATTCAG	This work
R2896	flg_ΔUTR_insertion_F1	GTGTCCATTGATTTCTTTCAGTTTCGGATCCGATTGTGCTCAATCTCATGGAG	This work
R2843	flg_ΔUTR_insertion_R2	CTTGCATGTCTGCAGGCCTCGAGATAGCTGTGCTTCTTGAC	This work
R2882	flgOFF_3nt subs_F2	GTTGCCTTTTTTTGTAATATAGGTTCTTCATTTTTTATTAATAAGC	This work
R2883	flgOFF_3nt subs_R1	GCTTATTAATAAAAAATGAAGAACCTATATTACAAAAAAAGGCAAC	This work
R2869	flgOFF_ΔRIR_F2	CTTTATAAAGTTGCCCTTCATTTTTTATTAATAAGC	This work
R2870	flgOFF_ΔRIR_R1	GCTTATTAATAAAAAATGAAGGGCAACTTTATAAAG	This work
R2884	flgOFF_Δ3nt_F2	TATTTCTTTATAAAGTTGCCTTTTGTAATATAGCAACTTCATTTTTTATTAATAAG	This work
R2885	flgOFF_Δ3nt_R1	AAAAAATGAAGTTGCTATATTACAAAAGGCAACTTTATAAAG	This work
R2888	flgON_3nt subs_F2	GAATAAAGAAGTCATTTTTTGTAATATAGGTTCTTCATTTTTTATTAATAAGC	This work

R2889	flgON_3nt subs_R1	GCTTATTAATAAAAAATGAAGAACCTATATTACAAAAAATGACTTCTTTATTC	This work
R2886	flgON_ΔRIR_F2	GTAATTAATTTGGATGAATAAAGAAGTCCTTCATTTTTTATTAATAAGC	This work
R2887	flgON_ΔRIR_R1	GCTTATTAATAAAAAATGAAGGACTTCTTTATTCATCCAAATTAATTAC	This work
R2890	flgON_Δ3nt_F2	GTAATTAATTTGGATGAATAAAGAAGTCTTTTGTAATATAGCAACTTC	This work
R2891	flgON_Δ3nt_R1	TAAAAAATGAAGTTGCTATATTACAAAAGACTTCTTTATTCATCCAAATTAATTAC	This work
R1512	CDR202_PflgBnew	GTTCAAGCATGCGATATATTGTACAAATAAAATTGAAATATATGG	[23]
R1611	CDR20291_0248CDSR	CAAGAATTCTTACCTCCCACTTATTATTGA	[23]

[a] Restriction sites used for cloning are underlined
