# Supplementary material for: Flagellum and toxin phase variation impacts intestinal colonization and disease development in a mouse model of Clostridioides difficile infection
Source: Gut Microbes. 2022 Feb 22;14(1):2038854. doi: 10.1080/19490976.2022.2038854 (PMC8890394; doi:10.1080/19490976.2022.2038854)
Supplement: Supplemental Material [file KGMI_A_2038854_SM5851.zip › supplementary/downloadFromZipFile.pdf]

SUPPLEMENTAL DATA

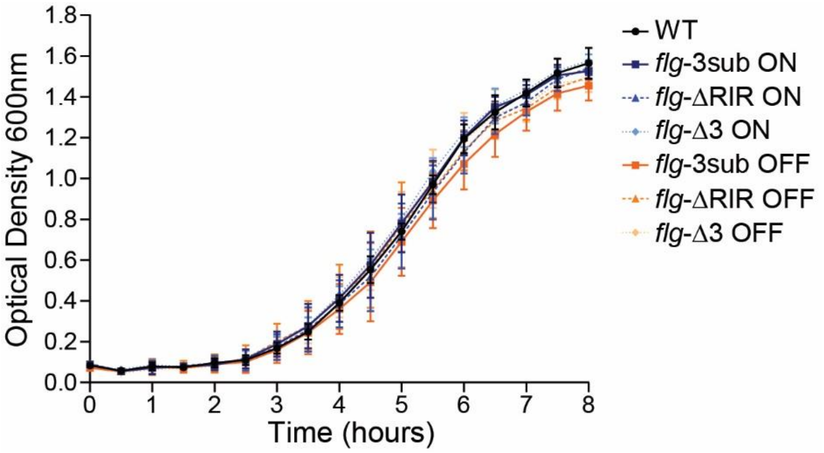

Figure S1. Mutations in *flg* RIR do not affect growth. Growth curves of WT, *flg* ON and OFF (3sub, ΔRIR, Δ3) mutants. Overnight cultures grown in TY medium were diluted 1:50 into BHIS broth. Optical density (OD_{600}) was measured every 30 minutes for 8 hours. Shown are the means and standard deviations for 6 biological replicates.

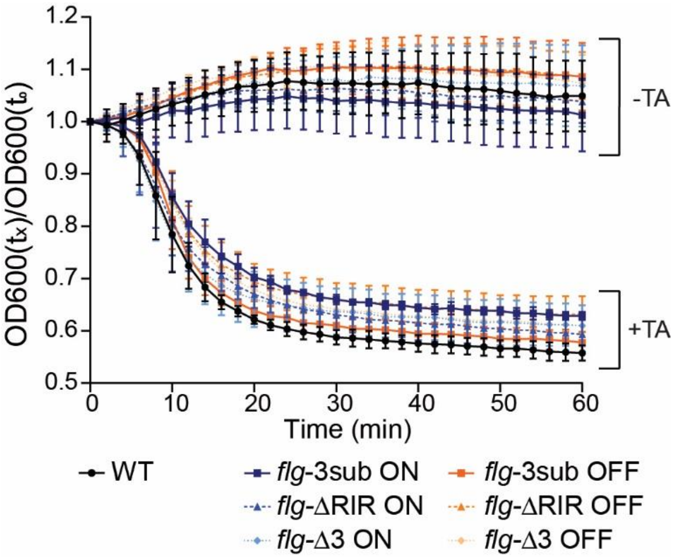

Figure S2. Mutations in the flagellar switch RIR do not affect germination. Purified spores of indicated strains were germinated in the presence of taurocholate (+) or in buffer without germinant as a control (-), and optical density (OD_{600}) was measured. Germination was plotted as the ratio of optical density (OD_{600}) at a given time point (t_x) versus initial OD_{600} (t_0). A representative germination plot of six independent experiments each consisting of 2 technical replicates is shown. Means and standard deviations are shown.

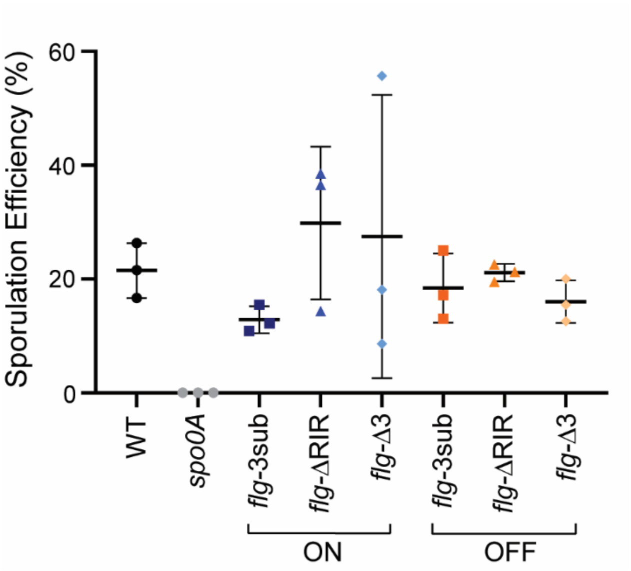

Figure S3. Mutations in the flagellar switch RIR do not affect sporulation. Sporulation efficiency was evaluated by ethanol resistance and calculated as the total number of spores divided by the total number of viable cells (spores plus vegetative). A sporulation-deficient *spo0A* mutant was included as a control. The means and standard deviations of three independent experiments are shown. n.d. – none detected. No significant differences from wildtype by one-way ANOVA other than for the *spo0A* control.

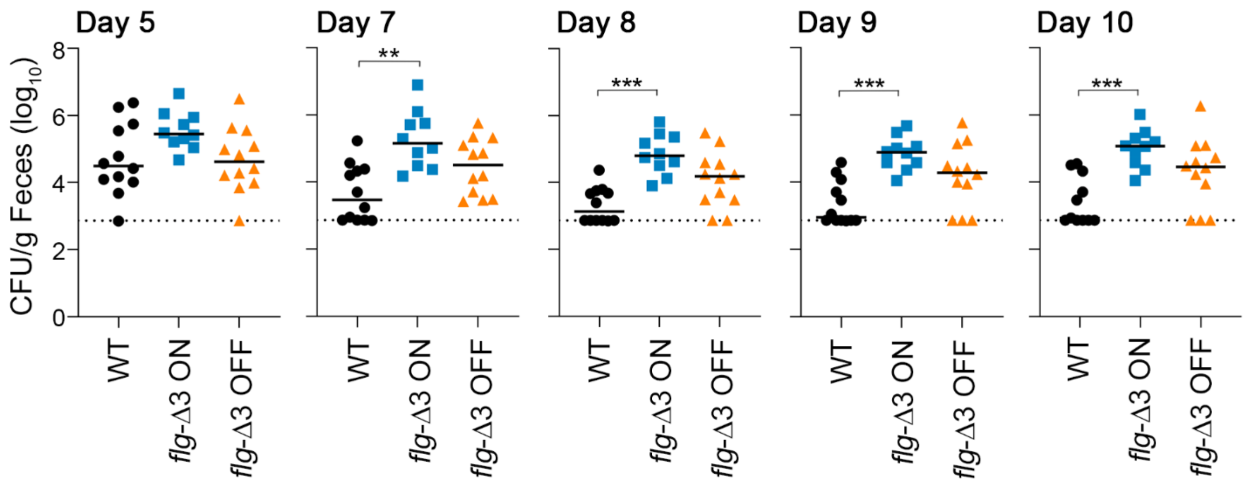

Figure S4. Bacterial load in feces collected from mice inoculated with WT, *flg*-Δ3 ON, and *flg*-Δ3 OFF. CFU enumerated in fecal samples collected every 24 hours p.i., with data in Figure 4A separated by day. Day 6 data are shown in Figure 4B. Two independent experiments with n = 6 (3 male, 3 female) were done and the data combined for n = 12. Bars indicate the medians, and dotted lines represent the limit of detection. No CFU were detected in feces of mock-inoculated mice. ***p*<0.01, ****p*<0.001, by Kruskal-Wallis test with Dunn's post-test comparing all strains.

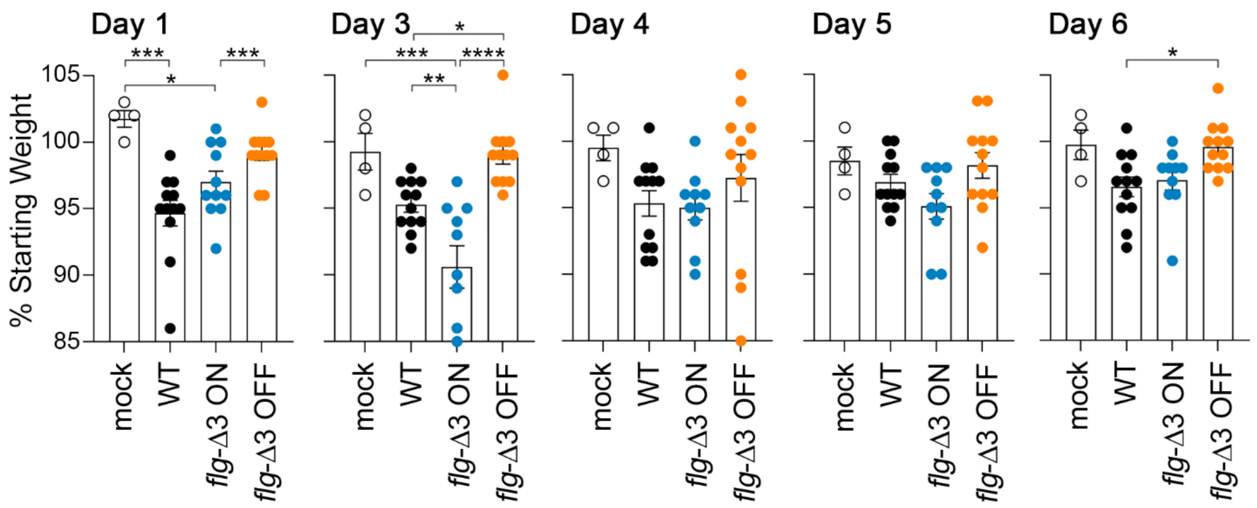

Figure S5. The *flg*-Δ3 ON mutant elicits increased weight loss in mice. Animal weights measured every 24 hours post-inoculation expressed as a percentage of the mouse's starting weight at day 0, with data from Figure 4C separated by day; day 2 data are shown in Figure 4D. Two independent experiments with n = 6 (3 male, 3 female) were done and the data combined for n = 12. Symbols represent values from individual animals, and bars indicate the means and standard error. **p*<0.05, ***p*<0.01, ****p*<0.001 by one-way ANOVA with Tukey's post-test comparing all strains.
